# Two new *Paraparatrechina* (Hymenoptera, Formicidae) species from the Seychelles, with notes on the hypogaeic *weissi* species-group

**DOI:** 10.3897/zookeys.414.7542

**Published:** 2014-06-06

**Authors:** John S. LaPolla, Brian L. Fisher

**Affiliations:** 1 Department of Biological Sciences, Towson University, Towson, Maryland 21252, USA; 2 Department of Entomology, 55 Music Concourse Drive, California Academy of Sciences Golden Gate Park, San Francisco, CA 94118, USA

**Keywords:** Ants, Formicinae, *Prenolepis* genus-group, Seychelles, new species

## Abstract

Recent survey work in the Seychelles has revealed two new species of *Paraparatrechina* that are here described: *P. illusio* **sp. n.** and *P. luminella* **sp. n.** A revised key to the workers of *Paraparatrechina* for the Afrotropical and Malagasy regions is provided. The taxonomy of the hypogaeic *weissi* species-group is also reviewed in light of recent field collections. The species *P. sordida* is revived from synonymy and given new status (as a full species) and a discussion of the morphologically peculiar species-group is provided. With the description of the two species and the removal of another species from *weissi* synonomy there are now 16 *Paraparatrechina* species known from the Afrotropical and Malagasy regions.

## Introduction

The biology of the ant genus *Paraparatrechina* remains poorly known, but what is becoming clearer is that species diversity within the genus is certainly much higher than is currently recognized. LaPolla et al. (2010b) recently revised the Afrotropical and Malagasy species and found 8 new species (they found 13 total species within the two regions). While the Australasian species await taxonomic revision, preliminary data suggest that there are many undescribed species (S. Shattuck, pers. comm.). To emphasize this point of the genus having much higher species richness than is currently recognized, recent survey work in the Seychelles by BLF revealed two new species that were not included in LaPolla et al. (2010b). Here we describe those two species.

We also provide notes on a rather unusual group of *Paraparatrechina*, the Afrotropical *weissi* species-group (LaPolla 2004a; LaPolla et al. 2010a; LaPolla et al. 2010b). This group was last reviewed by LaPolla (2004a) and thought to contain two valid species (*Paraparatrechina bufona* and *Paraparatrechina weissi*). Recent collection work however in Uganda suggests that a third species, *Paraparatrechina sordida*, which is currently in synonymy with *Paraparatrechina weissi*, should be elevated to full species. Here we also discuss the taxonomic status of the *weissi* species-group and provide images for all three species.

## Materials and methods

Specimens examined for this study are deposited in the following institutions:

CASC California Academy of Sciences, San Francisco, CA, USA

MCZC Museum of Comparative Zoology, Cambridge, MA, USA

USNM National Museum of Natural History, Washington, DC, USA

All measurements were taken at 80× power with a Leica M125 microscope using an orthogonal pair of micrometers, recorded to the nearest 0.001 mm, and rounded to two decimal places for presentation. When more than one specimen was measured, minimum and maximum measurements and indices are presented. All measurements are given in millimeters. Digital color images were created using a Leica DFC425 digital camera. Leica Application Suite software (ver. 3.8) was used for images. Each imaged specimen is uniquely identified with a specimen-level unique identifier (e.g. CASENT0003099).

Morphological terminology for measurements and indices employed throughout are defined (following LaPolla et al. 2011a, b) as:

EL (Eye Length): maximum length of compound eye in full-face view.

GL (Gaster Length): the length of the gaster in lateral view from the anteriormost point of the first gastral segment (third abdominal segment) to the posteriormost point.

HL (Head Length): the length of the head proper, excluding the mandibles; measured in full-face view from the midpoint of the anterior clypeal margin to a line drawn across the posterior margin from its highest points.

HW (Head Width): the maximum width of the head in full-face view.

PW (Pronotal Width): the maximum width of the pronotum in dorsal view.

SL (Scape Length): the maximum length of the antennal scape excluding the condylar bulb.

TL (Total Length): HL+WL+GL

WL (Weber’s Length): in lateral view, the distance from the posteriormost border of the metapleural lobe to the anteriormost border of the pronotum, excluding the neck.

CI (Cephalic Index): (HW/HL) × 100

REL (Relative Eye Length Index): (EL/HL) × 100

SI (Scape Index): (SL/HW) × 100

### Key to *Paraparatrechina* workers in the Afrotropical and Malagasy Regions (modified from LaPolla et al. 2010b)

**Table:** 

1	Eyes small relative to head length (REL ≤ 16)	2
–	Eyes medium to large relative to head length (REL ≥ 17)	6
2	Eyes consisting of less than 10 facets; polymorphic, with clearly expressed major caste; Equatorial Africa; *weissi* species-group	3
–	Eyes consisting of more than 10 facets; monomorphic; Madagascar	5
3	Scapes with numerous erect macrosetae; mesosoma with numerous erect macrosetae scattered across each segment (especially abundant on pronotum and mesonotum)	*Paraparatrechina bufona*
–	Scapes without erect macrosetae; few macrosetae on mesosoma (typically 2 on prontoum; 1 on mesonotum and 1 on propodeum)	4
4	Metanotal groove strongly impressed; head without paired macrosetae medially from posterior margin towards clypeus; no macrosetae on posterior margin	*Paraparatrechina sordida* stat. n. & rev.
–	Metanotal groove not strongly impressed; head with paired macrosetae medially from posterior margin towards clypeus; at least four macrosetae on posterior margin	*Paraparatrechina weissi*
5	Scape with decumbent pubescence; scapes surpass posterior margin by approximately length of the first 3–4 funicular segments	*Paraparatrechina myops*
–	Scape with appressed pubescence; scapes surpass posterior margin by approximately length of the first 2–3 funicular segments	*Paraparatrechina ocellatula*
6	Mesosoma elongate in lateral view, with pronotum gently rising towards mesonotum	7
–	Mesosoma compact in lateral view, with pronotum steeply rising towards mesonotum	9
7	Propodeum with a short, angular dorsal face, and a long declivitous face; scape length <0.6 mm; Madagascar	*Paraparatrechina glabra*
–	Propodeum with rounded dorsal face, not conspicuously longer than declivitous face; scape length >0.6 mm; Equatorial Africa	8
8	Scape length > 0.72 mm; tibiae same brown color as mesosoma; protrochanter brown as in mesosoma, but meso/metatrochanters may be lighter brown; mandibles and antennae typically same brown color as head	*Paraparatrechina splendida*
–	Scape length < 0.72 mm; tibiae whitish to brownish-yellow; all trochanters white; mandibles and antennae yellowish-brown, contrasting with brown head	*Paraparatrechina concinnata*
9	Mesosomal dorsum (primarily pronotum and mesonotum) much lighter (typically yellow against brown or white against dark brown) than remainder of mesosoma	10
–	Mesosomal dorsum not much lighter than remainder of mesosoma	11
10	Dorsum of gaster with a distinctly yellow to white patch of color contrasting with remainder of gaster; Seychelles	*Paraparatrechina luminella* sp. n.
–	Dorsum of gaster solid dark brown in color; West Africa	*Paraparatrechina albipes*
11	Gaster brown, conspicuously contrasting with yellow head and mesosoma	*Paraparatrechina umbranatis*
–	Gaster brownish-yellow to yellow, not conspicuously contrasting with head and mesosoma	11
12	Dark brown species, with conspicuously lighter colored, contrasting antennae and legs	*Paraparatrechina brunnella*
–	Yellow species, with antennae and legs same color as remainder of body	13
13	Smaller species (HL & SL < 0.4 mm)	*Paraparatrechina gnoma*
–	Larger species (HL & SL > 0.4 mm)	14
14	Scapes with appressed pubescence	*Paraparatrechina oreias*
–	Scapes with decumbent pubescence	15
15	Short, decumbent pubescence covers head, especially lateroposteriorly, where it is longer than remainder of head; pubescence on gaster longer, slightly decumbent, giving an “unkempt” appearance; West Africa	*Paraparatrechina subtilis*
–	Short, decumbent pubescence present lateroanteriorly around eyes; pubescence on gaster shorter, tightly appressed to gaster, with pubescence appearing in neat rows with a silky appearance; Seychelles	*Paraparatrechina illusio* sp. n.

## New species accounts

### Paraparatrechina illusio sp. n.

http://zoobank.org/94A7F5B0-FAF1-477D-B697-71148BD9F5D4

http://species-id.net/wiki/Paraparatrechina_illusio

Figs 1 – 9

#### Holotype worker.

SEYCHELLES: Praslin Island, 280m, 4.34725°S, 55.74743°E, 6.ii.2010, mixed palm forest, on low vegetation, B.L. Fisher et al. CASENT0159099 (CASC); 5 paratype workers, SEYCHELLES: Conception Island, 65 m, 4.66311°S, 55.36821°E, 12.ii.2010, mixed forest, B.L. Fisher et al. CASENT0160297 (CASC), CASENT0160331 (CASC), CASENT0160622 (USNM), CASENT0160625 (CASC), CASENT0160650 (CASC); paratype worker, SEYCHELLES: Curieuse Island, 5 m, 4.28364°S, 55.7269°E, 4.ii.2010, coastal scrub, B.L. Fisher et al. CASENT0160122 (CASC); paratype worker, SEYCHELLES: Praslin Island, Newcome, 130m, 4.390°S, 55.6926°E, 6.ii.2010, palm forest, B.L. Fisher et al. CASENT0158991 (USNM); paratype worker, SEYCHELLES: Sillhouette Island, 20 m, 4.49076°S, 55.25341°E, 21.i.2010, coastal scrub, B.L. Fisher et al., CASENT0159838 (CASC).

#### Worker diagnosis.

Short, decumbent pubescence present lateroanteriorly around eyes; pubescence on gaster shorter, tightly appressed to gaster, with pubescence appearing in neat rows with a silky appearance.

Compare with: *Paraparatrechina gnoma*, *Paraparatrechina oreias*, and *Paraparatrechina subtilis*.

#### Worker.

Measurements (n=8) TL: 1.20–1.67; HW: 0.35–0.38; HL: 0.39–0.42; EL: 0.10–0.11; SL: 0.37–0.40; PW: 0.23–0.26; WL: 0.41–0.44; GL: 0.39–0.81

*Indices*: CI: 86-95; REL: 25-27; SI: 101-112.

Yellow to brownish-yellow; legs and antennae lighter; short, decumbent pubescence present lateroanteriorly around eyes; pubescence on gaster short, tightly appressed to gaster, with pubescence appearing in neat rows with a silky appearance. Head subquadrate with nearly straight posterior margin; scapes surpass posterior margin by first 2-3 funicular segments; three ocelli apparent. Mesosoma compact with steeply rising pronotum in lateral view; metanotal area indistinct, only slightly impressed; propodeum with short, flat dorsal face and much longer, steep declivitous face.

**Figures 1–3.**
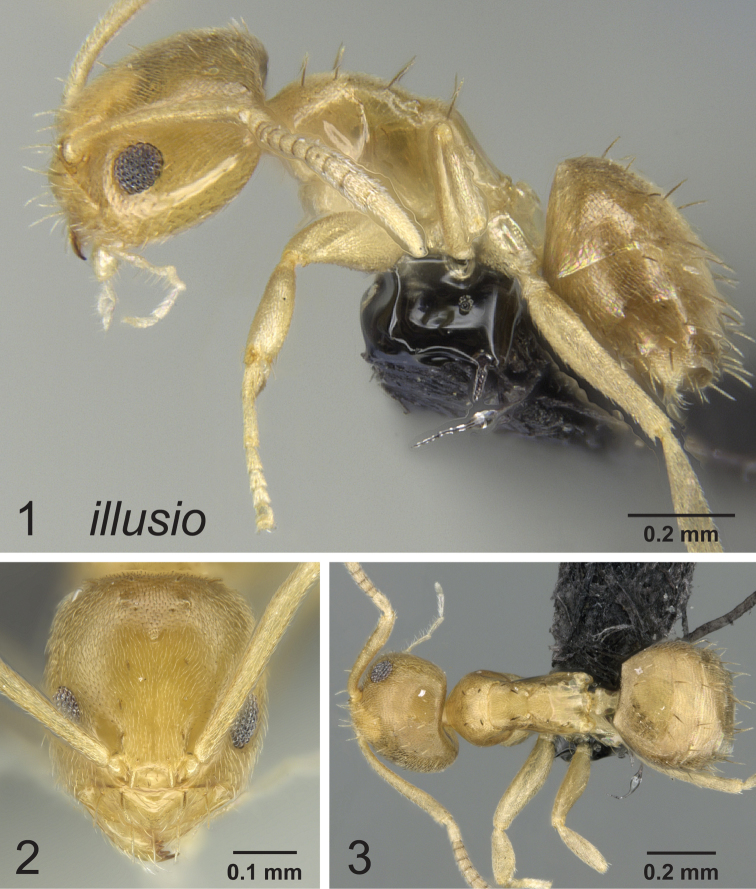
Lateral, full face and dorsal view of body. *Paraparatrechina illusio* holotype worker CASENT0159099.

#### Queen.

Measurements (n=3) TL: 2.92–3.23; HL: 0.54–0.55; HW: 0.58–0.61; EL: 0.20–0.21; SL: 0.53–0.53; PW: 0.62–0.66; WL: 0.97–1.03; GL: 1.34–1.70

*Indices*: CI: 110-111; REL: 36-38; SI: 86-88

As in worker, with modifications expected for queen caste and the following differences:

1. Pubescence distinctly across head and mesosoma.
2. Gaster darker (yellowish-brown) than remainder of body.

**Figures 4–6.**
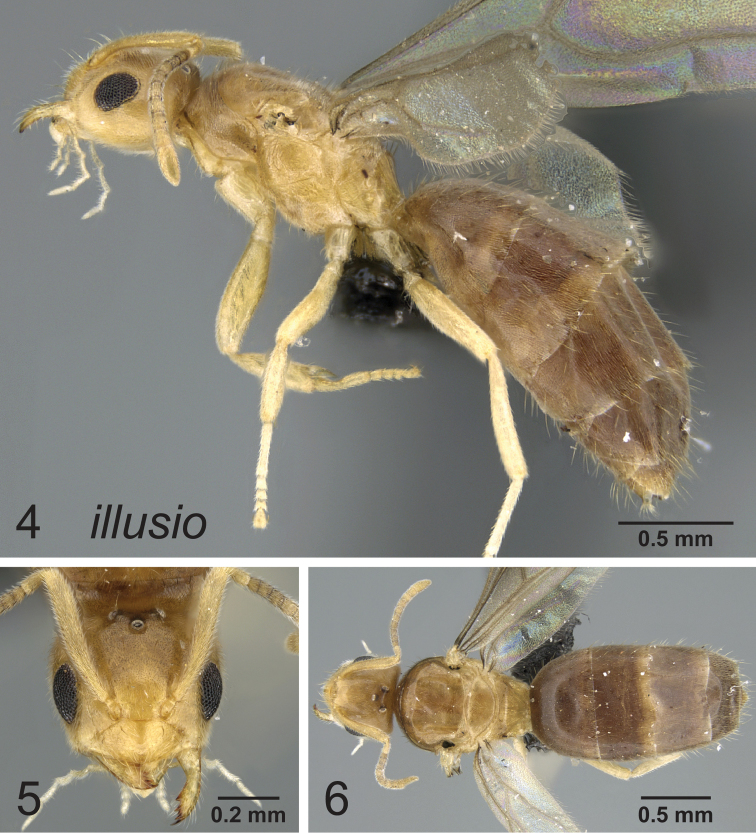
Lateral, full face and dorsal view of body. *Paraparatrechina illusio* queen CASENT0160097.

#### Male.

Measurements (n=1) TL: 1.55; HL: 0.33; HW: 0.38; EL: 0.18; SL: 0.31; PW: 0.29; WL: 0.54; GL: 0.68

*Indices*: CI: 113; REL: 53; SI: 81

Head brown, with bulging large eyes that occupy most of the lateral region of the head; head slightly broader than long. Palps distinctly lighter than head in color. A dense layer of pubescence covers head, with scattered erect setae along mid-region, posterior margin and clypeus. Scapes surpass posterior margin by about length of the first 2 funicular segments; antennae 13-segmented. Mandible with apical tooth and an indistinct basal angle. Mesosoma same color as head; pronotum short and collar-like; mesonotum large, rounded anteriorly, overarching pronotum; mesosoma dorsum flat, with erect setae. Gaster slightly ligher brown than head and mesosoma, covered with pubescence and erect setae. Parameres relatively broad then with a steep angle towards last third of paramere length; last third of paramere thinner and elongated with rounded apex; paramere with scattered erect setae.

**Figures 7–9.**
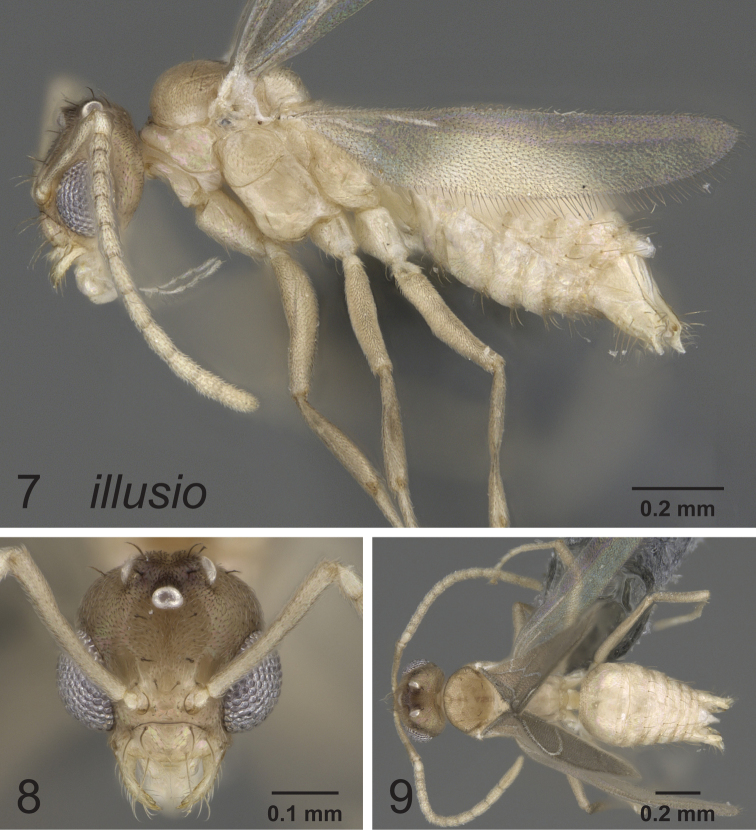
Lateral, full face and dorsal view of body. *Paraparatrechina illusio* male CASENT0914145.

#### Notes.

This species falls into the small, yellow *Paraparatrechina* worker phenotype range (typically workers of these species vary by only slight difference in setation and color tones of yellow and brownish-yellow) and therefore identification of the species can be difficult. It is morphological similar to three Aftrotropical species: *Paraparatrechina gnoma*, *Paraparatrechina oreias*, and *Paraparatrechina subtilis*. In practice, a relatively straight forward, non-morphological way, to separate *Paraparatrechina illusio* is it is only known from the Seychelles, while the remaining three species are from West and Central Africa. Morphologically, it differs from *Paraparatrechina gnoma* in being slightly larger overall and in coloration (*Paraparatrechina gnoma* is brownish-yellow with lighter yellow patches). From *Paraparatrechina oreias* the main difference also lies in color. The gaster of *Paraparatrechina oreias* is brownish-yellow, contrasting slightly with the remainder of the body. The metanotal area of *Paraparatrechina oreias* is also more distinctly defined than is seen in *Paraparatrechina illusio*. From *Paraparatrechina subtilis*, the difference is in the pubescence. Whereas the pubescence on the head of *Paraparatrechina subtilis* is decumbent throughout, on *Paraparatrechina illusio* it is only decumbent lateroanteriorly around eyes. Additionally the gastral pubescence is different between the two: in *Paraparatrechina subtilis* it is longer and slightly decumbent, contrasting with the shorter, tightly appressed pubescence observed in *Paraparatrechina illusio*.

### Paraparatrechina luminella sp. n.

http://zoobank.org/4A4A4FCF-DFDD-462E-AB98-C707B61F7A31

http://species-id.net/wiki/Paraparatrechina_luminella

Figs 10 –15

#### Holotype worker.

SEYCHELLES: Silhouette Island, above Jardin Marron on crest to Mont Plaisir and Pot à Eau, 520m, 4.4867°S, 55.2341°E, 20.i.2010, forest, rotten log, B.L.Fisher et al. CASENT0159693 (CASC); paratype worker, same locality as holotype (USNM); 2 paratype workers, SEYCHELLES: Mahé Island, Mont Copolia, 520m, 4.65121°S, 55.45835°E, 8.ii.2010, forest, sifted litter, B.L.Fisher et al. CASENT0159361 (CASC), CASENT0159373 (CASC); paratype worker, SEYCHELLES: Mahé Island, Le Niol, 345m, 4.63067°S, 55.43159°E, 11.ii.2010, tree plantation, rotten log, B.L.Fisher et al. CASENT0159051 (CASC); paratype worker, SEYCHELLES: Mahé Island, Casse Dent, Morne Seychellois National Park, 465 m, 4.65284°S, 55.43735°E, 11.ii.2010, mixed forest, under rootmat, B.L.Fisher et al. CASENT0145383 (CASC); paratype worker, SEYCHELLES: Silhouette Island, below Mont Cocos Marrons, 320m, 4.50248°S, 55.24395°E, 21.i.2010, forest, under rootmat, B.L.Fisher et al. CASENT0158936 (CASC); paratype worker, SEYCHELLES: Silhouette Island, on ridge toward Pot à Eau, 600m, 4.48213°S, 55.23408°E, 22.i.2010, moist rainforest, rotten log, B.L.Fisher et al. CASENT0158939 (CASC); paratype worker, SEYCHELLES: Silhouette Island, Jardin Marron, 395m, 4.48636°S, 55.23627°E, 27.i.2010, non-native forest, on low vegetation, B.L.Fisher et al. CASENT0159308 (CASC); paratype worker, SEYCHELLES: Silhouette Island, on ridge toward Mont Corgat, 445m, 4.49537°S, 55.23946°E, 25.i.2010, forest, ground nest, B.L.Fisher et al. CASENT0159944 (CASC); paratype worker & queen, SEYCHELLES: Silhouette Island, ridge from Mont Corgat to Mont Cocos Marron, 455m, 4.50126°S, 55.23985°E, 24.i.2010, forest, rotten log, B.L.Fisher et al. CASENT0159905 (USMN).

#### Worker diagnosis.

Very distinct patches of lighter areas (ranging from yellow to white) on pronotum (that can extend onto mesonotum) and gastral tergites (typically from posterior of T1 through anterior portion of T4.)

Compare with: *Paraparatrechina albipes*.

#### Worker.

Measurements (n=8) TL: 1.37–1.58; HW: 0.38–0.46; HL: 0.44–0.47; EL: 0.11–0.13; SL: 0.45–0.50; PW: 0.20–0.29; WL: 0.46–0.52; GL: 0.46–0.67

*Indices*: CI: 85–98; REL: 25–28; SI: 108–124;

Overall brown with patches of yellow to white; lighter area medially between eyes and above torulae; distinct patches of lighter areas (ranging from yellow to white) on pronotum (that typically extends onto mesonotum and occasionally onto dorsal face of propodeum) and gastral tergites (typically from posterior of T1 through anterior portion of T4; scapes proximally more brown becoming yellow to whitish midlength, lightening to white through apex of funiculus; procoxae golden yellow, meso/metacoxae and trochanters white; femur golden yellow then remainder of leg light yellow to white; body covered in dense, appressed pubescence; macrosetae placement as is typical in *Paraparatrechina*. Head ovate with nearly straight posterior margin; scapes surpass posterior margin by first 2-3 funicular segments; three ocelli apparent. Mesosoma compact with steeply rising pronotum in lateral view; mesonotum and metanotal area short; metanotal area indistinct, only slightly impressed; propodeum with short, flat dorsal face and much longer, steep declivitous face.

**Figures 10–12.**
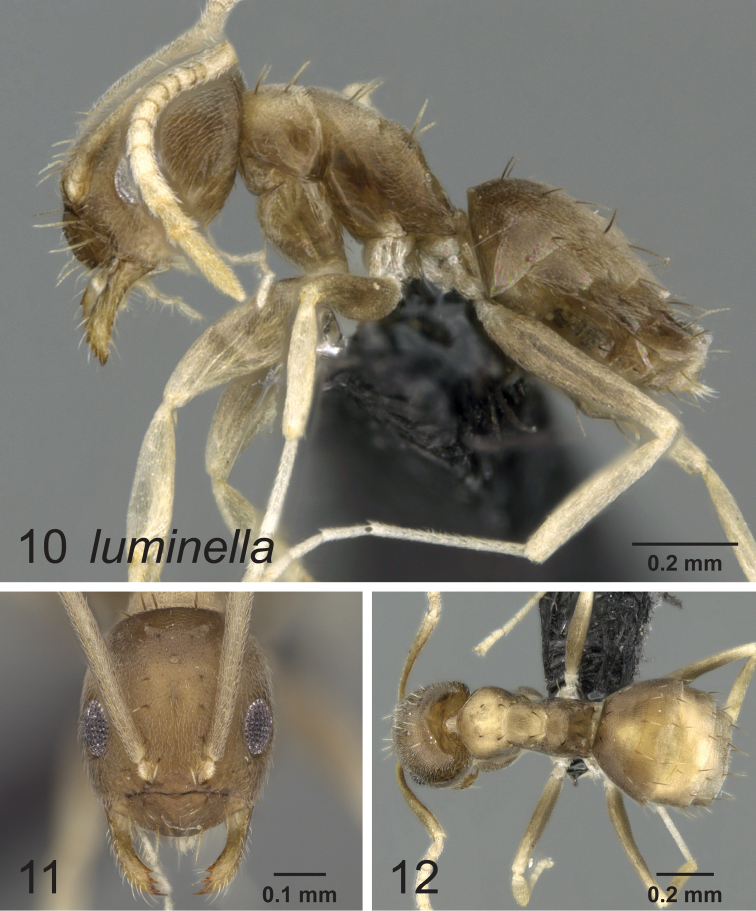
Lateral, full face and dorsal view of body. *Paraparatrechina luminella* worker CASENT0160868.

#### Queen.

Measurements (n=3) TL: 3.34–3.75; HL: 0.61–0.63; HW: 0.66–0.69; EL: 0.25–0.26; SL: 0.61–0.62; PW: 0.66–0.74; WL: 1.13–1.22; GL: 1.52–2.02

*Indices*: CI: 104–111; REL: 39–41; SI: 91–92

As in worker, with modifications expected for queen caste and the following differences:

1. Coloration overall more brown than in worker, with no distinct yellow to white patches on mesosoma or gaster; lighter antennae and legs.
2. Head subcordate.
3. Legs generally as in worker, except coxae brown.

**Figures 13–15.**
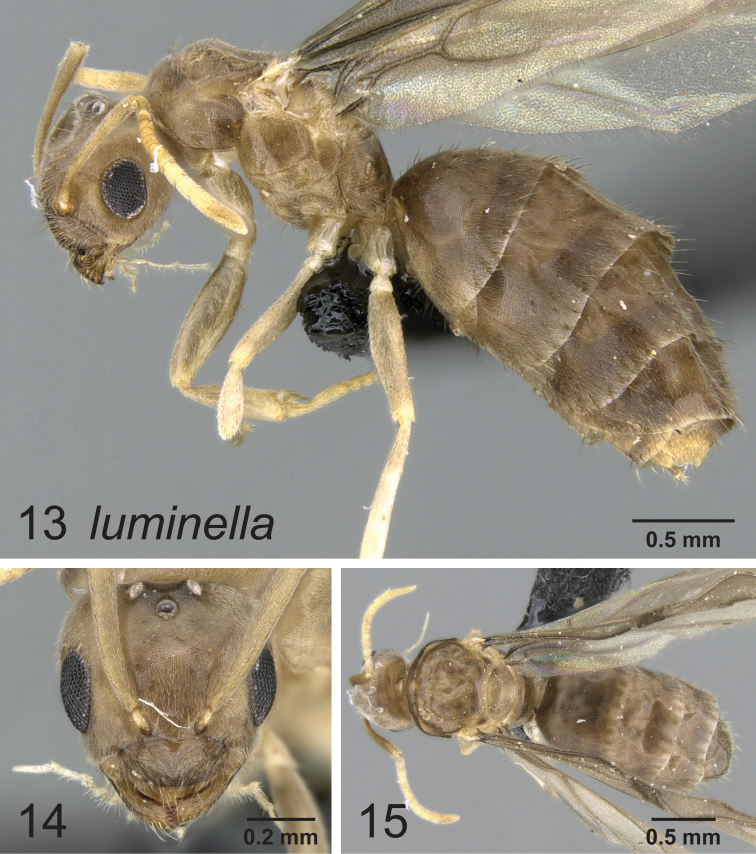
Lateral, full face and dorsal view of body. *Paraparatrechina luminella* queen CASENT0159313.

#### Notes.

The coloration pattern seen in the worker of *Paraparatrechina luminella* is very distinct and is unlike any pattern seen in other species from either the Afrotropical or Malagasy regions. The most similar coloration pattern among *Paraparatrechina* is seen in *Paraparatrechina albipes*, in which workers typically have a light patch of whitish coloration on the posterior pronotum and mesonotum that contrasts with the generally overall dark brown color of the body. Whether this is indicative of a close relationship between these two species or is simply convergence is unclear.

### Synopsis of species in the *Paraparatrechina weissi* species-group

*Paraparatrechina bufona* (Wheeler, 1922)

*Paraparatrechina sordida* (Santschi, 1914), **stat. n. & rev.**

= *Paraparatrechina gowdeyi* (Wheeler, 1922)

= *Paraparatrechina bucculentus* (Wheeler, 1922)

*Paraparatrechina weissi* (Santschi, 1910)

= *Paraparatrechina bayonii* (Menozzi, 1924)

= *Paraparatrechina myersi* (Weber, 1943)

= *Paraparatrechina myersi occipitalis* (Weber & Anderson, 1950)

### Notes on the Afrotropical *weissi* species-group

**Figures 16–18.**
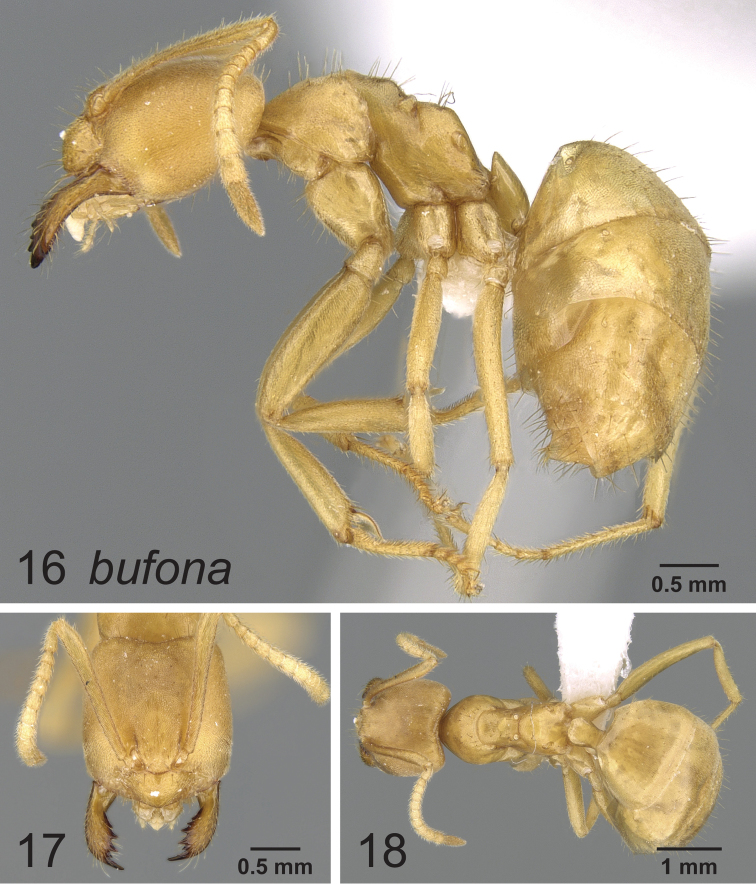
Lateral, full face and dorsal view of body. *Paraparatrechina bufona* worker CASENT0906212.

**Figures 19–21.**
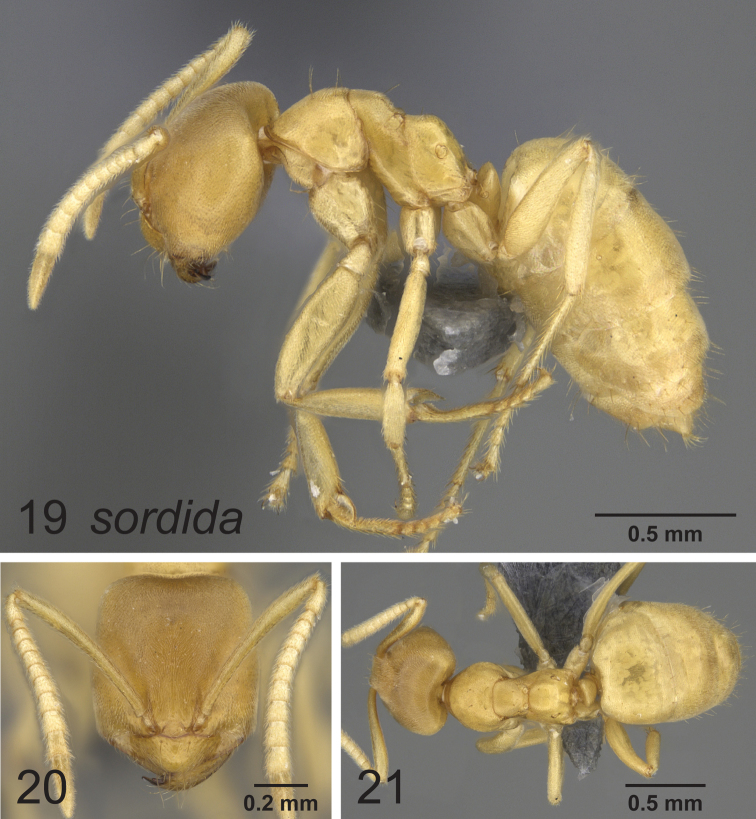
Lateral, full face and dorsal view of body. *Paraparatrechina sordida* worker CASENT0914143.

**Figures 22–24.**
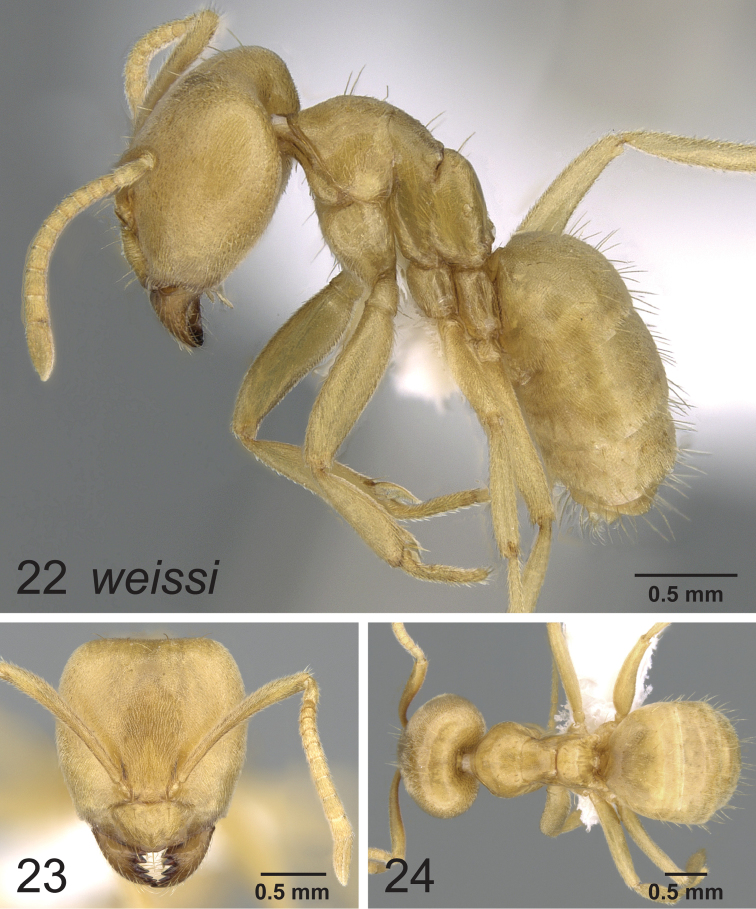
Lateral, full face and dorsal view of body. *Paraparatrechina weissi* worker CASENT0906210.

The now called *weissi* species-group was last reviewed by LaPolla (2004a), where they were considered to belong to the genus *Pseudolasius*. Later, LaPolla et al. (2010a) found based on molecular evidence from 5 genes that these morphologically peculiar species in fact belonged in *Paraparatrechina*. In retrospect, there was some, albeit at the time seemingly rather weak morphological support for the placement within *Paraparatrechina* such as: the short, angular dorsal propodeal face (Figs 16, 19, 22), and although obscured in *Paraparatrechina bufona* by the presence of several erect macrosetae on the mesosoma, the typical *Paraparatrechina* mesosomal macrosetae pattern of 2:1:1 (pronotum, mesonotum and propodeum) is present on all species. Nonetheless, superficially the *weissi* species-group does resemble *Pseudolasius*. What this certainly reflects is that the *weissi* species-group species have become hypogaeic and have convergently taken on the suite of morphological characters common among subterranean formicines (for example see LaPolla 2004b). Of further interest is that like *Pseudolasius* at least two species of the *weissi* species-group have evolved majors (*Paraparatrechina bufona* and *Paraparatrechina weissi*). All *Pseudolasius* (of which most, if not all species are hypogaeic) presumably have majors (LaPolla et al. 2010a). There appears to be selection occurring in the *Prenolepis* genus-group among those with a hypogaeic lifestyle for the evolution of majors. For instance, the ground-dwelling and presumably largely hypogaeic (based on the morphology of the workers) *Nylanderia amblyops* known from Madagascar appears to be the only species in that genus to have evolved majors. In *Euprenolepis* at least one species, *Euprenolepis procera*, has majors as well, although it is not hypogaeic (rather it appears to be nocturnal) (LaPolla 2009).

LaPolla (2004a) reviewed what would later be called the *weissi* species-group, and recognized only two valid species (*Paraparatrechina bufona* and *Paraparatrechina weissi*), the others of which were considered synonyms (all of *Paraparatrechina weissi*). The study was hindered in two ways: the lack of recent material from nest series and the confounding variable of worker polymorphism. Recent fieldwork (J. Longino, pers. comm.) however resulted in a small collection of specimens of two species of the *weissi* species-group in sympatry with each other: *Paraparatrechina weissi* and another species that was clearly not *Paraparatrechina bufona*. Upon comparison with type material, we determined that the specimens clearly belonged to the species named *Paraparatrechina weissi sordida* (here treated as a full species), previously synonymized under *Paraparatrechina weissi*. *Paraparatrechina sordida* differs from *Paraparatrechina weissi* and *Paraparatrechina bufona* in several ways. *Paraparatrechina bufona* is distinct because of the presence of many erect macrosetae across the scapes and mesosoma. The other two species *Paraparatrechina sordida* and *Paraparatrechina weissi* are more similar to each other but differ in that *Paraparatrechina sordida* possesses a strongly impressed metanotal groove, a head without paired macrosetae medially from the posterior margin towards the clypeus and has no macrosetae on the posterior margin of the head. We therefore propose a revived and new status of *Paraparatrechina sordida* as a full species. The synopsis of the *weissi* species-group provides a reinterpretation of the valid species and where the synonyms should properly be placed. These findings nicely demonstrate the continued need for collection in the very poorly sampled Afrotropical region.

## Supplementary Material

XML Treatment for Paraparatrechina illusio

XML Treatment for Paraparatrechina luminella
